# Failure to Genotype: A Cautionary Note on an Elusive loxP Sequence

**DOI:** 10.1371/journal.pone.0165012

**Published:** 2016-10-21

**Authors:** Johan Kreuger, Paul O’Callaghan

**Affiliations:** Department of Medical Cell Biology, Uppsala University, Uppsala, Sweden; University of Iowa, UNITED STATES

## Abstract

Here we report on a technical difficulty we encountered while optimizing genotyping strategies to identify mice derived from *Exoc3l2*^*tm1a(KOMP)Wtsi*^ embryonic stem cells obtained from the Knockout Mouse Project Repository. The *Exoc3l2*^*tm1a*(*KOMP*)*Wtsi*^ construct encodes a “knockout-first” design with loxP sites that confer conditional potential (KO^1st^). We designed primers that targeted wild-type sequences flanking the most downstream element of the construct, an 80 base pair synthetic loxP region, which BLAST alignment analysis reveals is an element common to over 10,000 conditional gene-targeting mouse models. As PCR products amplified from KO^1st^ and wild-type templates would have different lengths (and different mobility in an agarose gel) this strategy was designed to determine the zygosity of individual mice from a single PCR. In parallel we performed PCR with a primer specifically targeting the synthetic loxP sequence. Unexpectedly, while the latter strategy detected the synthetic loxP region and correctly genotyped KO^1st^ chimeric mice, the same individuals were genotyped as wild-type when using the primers that flanked the synthetic loxP region. We discuss the possibility that secondary DNA structures, formed due to the palindromic nature of the synthetic loxP region, may have caused the KO^1st^ template to elude the PCR when using primers that flanked this region. This brief report aims to raise awareness regarding this potential source of false-negative genotype results, particularly for those who are devising genotyping strategies for similarly engineered animal models.

## Introduction

Conditional gene knockout is a powerful tool for studying temporal and/or cell-type specific gene function. The Knockout Mouse Project (KOMP) Repository provides a comprehensive library of germline competent C57BL/6N embryonic stem (ES) cell clones encoding over 8,500 conditional gene-targeting constructs [1]. This has significantly increased the availability of mouse models and offered a standardized system for conducting loss-of-function studies.

**Fig 1:**
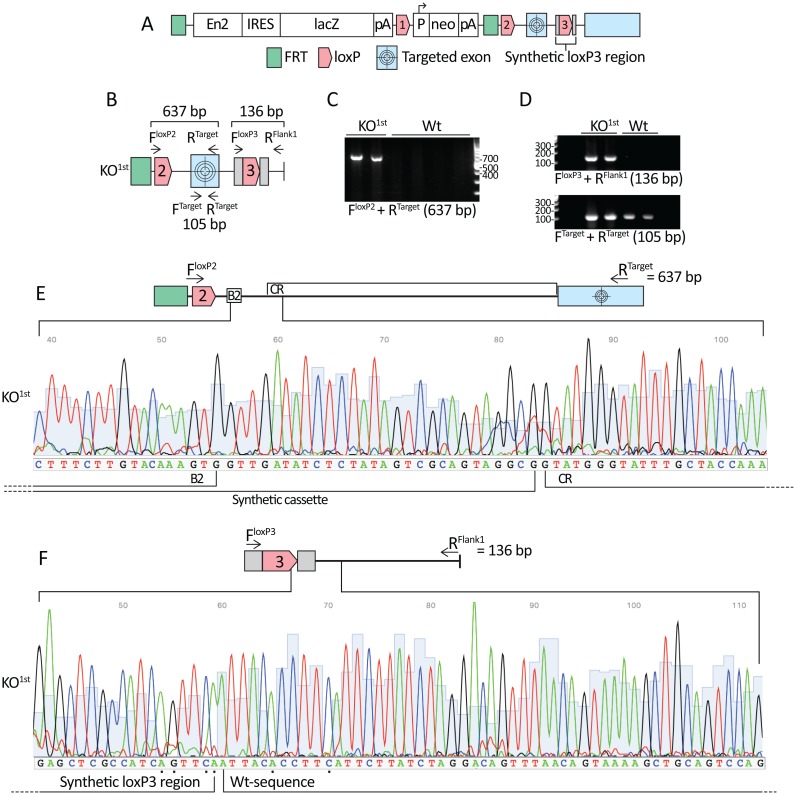
Genotyping *Exoc3l2*^*tm1a(KOMP)Wtsi*^ KO^1st^ chimeras with construct-specific primers. **A.** The *Exoc3l2*^*tm1a(KOMP)Wtsi*^ KO^1st^ construct with conditional potential. The targeted exon is flanked by a loxP (loxP2) and a synthetic loxP3 site. The final blue box represents the terminal exon of the *Exoc3l2* gene encoded by the construct. **B.** Combining primers designed to target loxP2 (F^loxP2^) and the target exon (R^Target^) was predicted to amplify a PCR product of 637 bp. The primer F^loxP3^ targeted the synthetic loxP3 region and in combination with the R^Flank1^ primer it was predicted to produce a 136 bp product from KO^1st^ templates. The F^Target^ and R^Target^ primers amplify a sequence of 105 bp from the targeted exon, common to all samples. **C.** PCR using the F^loxP2^ and R^Target^ primers confirmed the KO^1st^ genotype in DNA samples extracted from KO^1st^ chimera biopsies and amplified no product in samples extracted from Wt biopsies. **D.** PCR using the F^loxP3^ and R^Flank1^ primers with KO^1st^ and Wt biopsies also produced a PCR product close to the predicted size in KO^1st^ chimera samples and no product in Wt samples. PCR with F^Target^ and R^Target^ primers amplified a product close to the predicted size in all samples. **E.** Sanger sequencing of the PCR products from C. confirmed the KO^1st^ specific identity of the amplified product. **F.** Sanger sequencing of the 136 bp PCR product amplified using the F^loxP3^ and R^Flank1^ primers confirmed the correct position of the junction between the synthetic loxP3 sequence and the Wt sequence. Black dots were placed under bases of low quality, but the specific base peaks at these positions were identical to those predicted for this sequence. PCR was performed with an annealing temperature of 56°C and products were separated on 1% agarose gels containing GelRed stain. Each of the two KO^1st^ lanes in C and D represent PCR products derived from two distinct KO^1st^ chimeric mice. *Abbreviations*: B2: B2 Gateway (Gateway cloning strategy element) | CR: Critical region of *Exoc3l2* (identified by KOMP) | En2/SA: Engrailed-2/Splice acceptor | IRES: Internal ribosome entry site | lacZ: Lactose operon Z (β-galactosidase gene) | pA: SV40 polyadenylation site | P: Human beta actin promoter | neo: Neomycin resistance gene | KO^1st^: Knockout first chimera.

The ES cells available through the KOMP Repository are derived from a high throughput gene targeting strategy [1] that utilizes a knockout-first construct design [2] with conditional potential (KO^1st^). The construct (illustrated in Fig 1A) encodes a mouse En2 splice acceptor site, followed by an internal ribosome entry site (IRES), which precedes and promotes splicing to a lacZ insertion. This is followed by an SV40 polyadenylation sequence, which promotes termination of transcription (Fig 1A). These elements, together with a neomycin (neo) selection cassette, are incorporated into an intron upstream of a critical gene region and this approach has been successfully demonstrated to generate null alleles in mice [3, 4]. By crossing KO^1st^ homozygotes with mice expressing flippase it is possible to excise all elements flanked by the two flippase recognition target (FRT) sites. This reverts the mouse to a wild-type (Wt) state, but maintains conditional potential by preserving two loci of X-over P1 (loxP) sites flanking a critical gene region. Subsequent crossing with mice conditionally expressing Cre recombinase permits excision of the target sequence, which will again generate a null allele. Notably, while the Cre/loxP system of genetic engineering has been in use since the late eighties, its success as a conditional gene knock-out tool has lead to its integration into the design of model organisms derived using the revolutionary CRISPR/Cas9 technology [5].

An essential component of maintaining a colony of transgenic models is a reliable genotyping strategy. Polymerase chain reaction (PCR) with primers designed to selectively amplify products specific to the transgenic construct will distinguish KO^1st^ from Wt animals. However, additional reactions would be necessary to distinguish heterozygotes from homozygotes. Alternatively, zygosity can be determined using a single pair of primers designed to target Wt sequences that flank transgenic elements. This approach will produce products of two different lengths, depending on whether the DNA is amplified from Wt or KO^1st^ templates. Given that these products will have different mobility in agarose gel a heterozygote animal would be positive for both products, while Wt or homozygotes would be positive for single products of differing lengths. This approach is not always feasible given the specific design of certain transgenic constructs, but when possible it is a straightforward and informative method of genotyping.

The ability of nucleotide sequences to form secondary structures has been reported as a potential confounder of PCR [6]. Intrastrand base pairing can introduce stem and loop structures and this non-linear DNA conformation may exclude the template sequence from PCR. A single stranded loxP sequence consists of 34 bases of which the last 13 bases is a palindrome (or inverted repeat) of the first 13 bases. Consequently, base pairing between these regions can produce a hairpin loop structure.

Here we report on false negative genotype results for KO^1st^ mice derived from *Exoc3l2*^*tm1a(KOMP)Wtsi*^ ES cells. This occurred while attempting to amplify templates with primers that flanked a synthetic loxP region, consisting of 80 base pairs. We believe our experience is relevant and informative for those in the process of devising genotyping strategies for similarly engineered animal models.

## Materials and Methods

### Ethical permission

The ethical permission C222/11, relating to the animal experiments described herein, was granted by the Uppsala Animal Experiments Ethics Board (Uppsala Djurförsöksetiska Nämnd), Uppsala District Court, Uppsala, Sweden.

### Generation of chimeric *Exoc3l2*^*tm1a(KOMP)Wtsi*^ mice

The *Exoc3l2*^*tm1a(KOMP)Wtsi*^ ES cell clone EPD0331_5_B03 was obtained from the Knockout Mouse Project (KOMP) Repository (The Knockout Mouse Project, Mouse Biology Program, University of California, Davis, CA, USA). The *Exoc3l2* targeting project is archived at the KOMP Repository under the identifier CSD46682. These ES cells are of the C57BL/6N-A/a strain and the parental ES cell line JM8A1.N3 [7]. The KOMP Repository confirmed the *Exoc3l2*^*tm1a(KOMP)Wtsi*^ genotype of the ES cells by performing long-range PCR to determine that the *Exoc3l2* gene was correctly targeted; Taqman PCR to establish that only one vector was inserted in the genome; and short range PCR to confirm the presence of the synthetic loxP3 region. Chimeric mice (KO^1st^) were generated by injection of *Exoc3l2*^*tm1a(KOMP)Wtsi*^ ES cells into C57BL/6 blastocysts and transplanted into pseudopregnant foster females using the services of the Karolinska Institute, Karolinska Center for Transgene Technologies, Huddinge, Sweden. Chimeras were initially selected based on coat color and housed at the National Veterinary Institution, Uppsala, Sweden. Biopsies (tail or ear clippings) were obtained for genotyping. The F1 offspring analyzed in this study were generated by crossing one male and one female KO^1st^ chimera.

### DNA extraction from biopsies

Biopsies (approximately 1 mm^3^) were suspended in DNA extraction buffer [Modified Gitschier Buffer (67 mM Tris/HCl pH 8.8, 0.166 mM (NH_4_)2SO_4_, 6.5 mM MgCl_2_); 1.0% β-mercaptoethanol; 0.5% Triton X 100] and heat inactivated (95°C, 5 mins). The samples were chilled on ice and Proteinase K (Sigma-Aldrich, Stockholm, Sweden; 20 μg/sample) was added. Samples were digested at 55°C for 1 h with shaking following which Proteinase K was inactivated by heating (95°C, 10 mins). Samples were centrifuged (13,000 x rpm, 1 min) and the clear supernatants were collected and stored at -20°C. To isolate brain samples, mice were euthanized by cervical dislocation after being anaesthetized with isoflurane (Forene^®^; Abbott Scandinavia Ab, Solna, Sweden). DNA was extracted from brain homogenates using the spin-column based DNeasy^®^ Blood & Tissue Kit (Qiagen, Sollentuna, Sweden), according to the manufacturers instructions.

### Primer design

The *Exoc3l2*^*tm1a(KOMP)Wtsi*^ construct sequence is curated at the NCBI GenBank as JN946244.1 and the wild-type *Exoc3l2* gene sequence has the Gene ID: 74463. Primers were designed to target regions of interest in these sequences using the NCBI primer-BLAST tool. Oligonucleotide primer sequences were synthesized by Life Technologies (Thermo Fisher Scientific, Uppsala, Sweden). Primer sequences are presented in Table 1.

**Table 1:** Primer names, sequences and predicted PCR product sizes.

				Predicted product (bp)	
Primer name	Positive strand primer sequence	Primer name	Negative strand primer sequence	KO1st	Wt
F^loxP2^	TCGTCGAGATAACTTCGTATAGCA	R^Target^	GTAGTCCCGCACCAGCAC	637	/
F^loxP3^	GAGATGGCGCAACGCAATTAAT	R^Flank1^	TGGCTGGACTGCAGCTTTTA	136	/
F^Target^	CAGGCTTATTGGCTTGACCA	R^Target^	GTAGTCCCGCACCAGCAC	105	105
F^Flank1^	GTGTCTGACGGCTGTTTCCT	R^Flank1^	TGGCTGGACTGCAGCTTTTA	453	386
F^Flank2^	CAGGGCTCGCAAACAAACAA	R^Flank2^	AGATCTGCTGGTCTGCATTCAA	689	622
F^Flank3^	ATCTAGGTGGGCCACTTCCT	R^Flank3^	CAGATCTGCTGGTCTGCATTC	527	460
F^Flank4^	TTCCTTGTCTGCTGCCTACG	R^Flank4^	TCAGATCTGCTGGTCTGCATT	496	429
F^Flank5^	TTCCTTGTCTGCTGCCTACG	R^Flank5^	TGGCTGGACTGCAGCTTTTA	178	111

Primers were designed using the NCBI primer-BLAST webserver and the *Exoc3l2*^*tm1a(KOMP)Wtsi*^ KO^1st^ construct sequence, curated at NCBI GenBank as JN946244.1. Note that the primers annotated as F^Flank5^ and R^Flank5^ are simply a new pair combined from the primers F^Flank4^ and R^Flank1^.

### Polymerase chain reaction (PCR)

PCR was carried out with AmpliTaq Gold^®^ DNA polymerase with Buffer II and MgCl_2_ (Thermo Fisher Scientific). Taq polymerase and Buffer II were added as per the manufacturers instructions; additionally, one PCR volume contained 0.5 mM MgCl_2_, 5% dimethyl sulfoxide (DMSO), 1 M N,N,N-trimethylglycine (betaine), 0.1 μM dNTPs, 0.5 μM forward primer, 0.5 μM reverse primer, and 1.5 μl of sample DNA (1 μg/μl). The PCR was performed on a MiniOpticon PCR System (BioRad Laboratories, Solna, Sweden) or a 2720 Thermal Cycler (Applied Biosystems, Thermo Fisher Scientific) with the following protocol: one cycle of 95°C for 3 min; 39 cycles of 95°C for 30 sec; 56, 60, 65 or 70°C for 30 sec; 72°C for 1 min; and one cycle of 72°C for 7 min. PCR products and the GeneRuler 100 bp or 1 kb DNA ladder (Thermo Fisher Scientific) were diluted in DNA Gel Loading Dye (6X; Thermo Fisher Scientific) and separated by electrophoresis on 1% or 2.5% agarose gels, prepared in 40 mM Tris, 20 mM acetate and 1 mM EDTA (TAE buffer) with 1‱ GelRed fluorescent DNA stain (Biotium, Hayward, CA, USA). Gels were scanned using a Gel Do EZ imaging system (BioRad Laboratories) or an Odyssey Fc Imaging System (LI-COR Biosciences, Cambridge, United Kingdom) and images were collected using the ImageLab Software (BioRad Laboratories) or Image Studio^™^ Software (LI-COR Biosciences).

### Sanger sequencing

Amplicons of interest were excised from agarose gels. DNA was extracted from the gel using a QIAquick Gel Extraction Kit (Qiagen) according to the manufacturers instructions for spin-columns. The concentrations of extracted DNA were determined using a NanoDrop 2000 (NanoDrop, Thermo Fisher Scientific). Sanger sequencing was conducted by the Uppsala Genome Center, Science For Life Laboratory, Uppsala, Sweden. Sequence data was viewed and presented using 4Peaks (A. Griekspoor and Tom Groothuis, Nuclobytes B. V., nucleobytes.com) and Adobe Illustrator software.

### DNA secondary structure analysis

The 80 base DNA sequence GAGATGGCGCAACGCAATTAATGATAACTTCGTATAGCATACATTATACGAAGTTATGGTCTGAGCTCGCCATCAGTTCA that comprises the synthetic loxP region of the Exoc3l2^tm1a(KOMP)Wtsi^ construct was submitted to the RNAstructure Webserver (http://rna.urmc.rochester.edu/RNAstructureWeb/) [8]. DNA was selected as the nucleic acid type and default settings were maintained for all other data options. The RNAstructure MaxExpect Result was downloaded and annotated using Adobe Illustrator software.

### Nucleotide BLAST analysis of the synthetic loxP region

Sequence alignment of the synthetic loxP region was performed using the Nucleotide Basic Local Alignment Search Tool (BLAST) hosted on the National Center for Biotechnology Information (NCBI) webserver (https://blast.ncbi.nlm.nih.gov/Blast.cgi) [9]. The 80 base DNA sequence GAGATGGCGCAACGCAATTAATGATAACTTCGTATAGCATACATTATACGAAGTTATGGTCTGAGCTCGCCATCAGTTCA was submitted. Under the ‘Choose Search Set’ section the nucleotide collection (nr/nt) database was selected and the search was limited to the organism *Mus musculus* (taxid: 10090). The search was optimized for ‘Highly similar sequences (megablast)’ in the ‘Program Selection’ options. The default algorithm parameters return a maximum of 100 aligned target sequences; this can be increased under the ‘General Parameters’ section, and was set at the maximum of 20,000 aligned target sequences. The ‘Word size’ (i.e. the length of the seed that initiates an alignment) was increased to 64. All of the results returned by this search are annotated in the format ‘*Mus musculus targeted KO-first*, *conditional ready*, *lacZ-tagged mutant allele “gene name” (KOMP or EUCOMM)*, *transgenic’*. A summary of the results (10,114 alignments) with accession identifiers for each of the transgenic mouse models that align is attached as S1 File.

## Results and Discussion

A robust and reliable genotyping strategy is essential for managing transgenic mouse colonies. The *Exoc3l2*^*tm1a(KOMP)Wtsi*^ KO^1st^ construct consists of three loxP sites (Fig 1A), the most downstream of which is the synthetic loxP3 region, which contains a centrally located loxP sequence (Fig 1A) that is flanked by two construct-specific 23 base pair (bp) sequences (Fig 1A, grey boxes). We designed a forward primer that targeted the loxP2 site of the KO^1st^ construct (F^loxP2^) and combined it with a reverse primer against a Wt sequence in the target exon (R^Target^) (Fig 1B), which was predicted to amplify a 637 bp PCR product in mice carrying a KO^1st^ allele. As all loxP sites are comprised of identical sequences, to achieve locus-specific detection of the loxP2 site the F^loxP2^ primer targeted the first sixteen bases of the loxP2 site and eight locus-specific bases upstream of that. To test the PCR reactions, chimeras derived from KO^1st^ ES cells were included as positive controls (KO^1st^) and Wt C57BL6 mice as negative controls (Wt). PCR using biopsies from KO^1st^ mice yielded a single product close to the predicted amplicon size for the combination of F^loxP2^ and R^Target^ primers, while no product was amplified in Wt mice (Fig 1C). The bands were excised and subjected to Sanger sequencing (using the F^loxP2^ primer), which confirmed the presence of KO^1st^ construct-specific elements (Fig 1E). A section of the sequencing results, including the B2-gateway sequence (B2) and the end of the synthetic cassette that precedes the sequence annotated as a critical region (CR), is presented in Fig 1E. We could also confirm that the synthetic loxP3 region was correctly located by designing a forward primer that targeted the synthetic loxP3 region (F^loxP3^) combined with the R^Flank1^ primer (Fig 1B), which produced the predicted 136 bp product in KO^1st^ samples and no product in Wt samples (Fig 1D, upper panel). Sanger sequencing (using the F^loxP3^ primer) confirmed that the junction between the synthetic loxP3 region and the Wt sequence was correctly postioned (Fig 1F). To demonstrate that DNA was effectively isolated from the biopsies, we also performed PCR using primers (F^Target^ and R^Target^) that amplify the targeted exon (Fig 1B), which is common to all genotypes (Fig 1D, lower panel).

**Fig 2:**
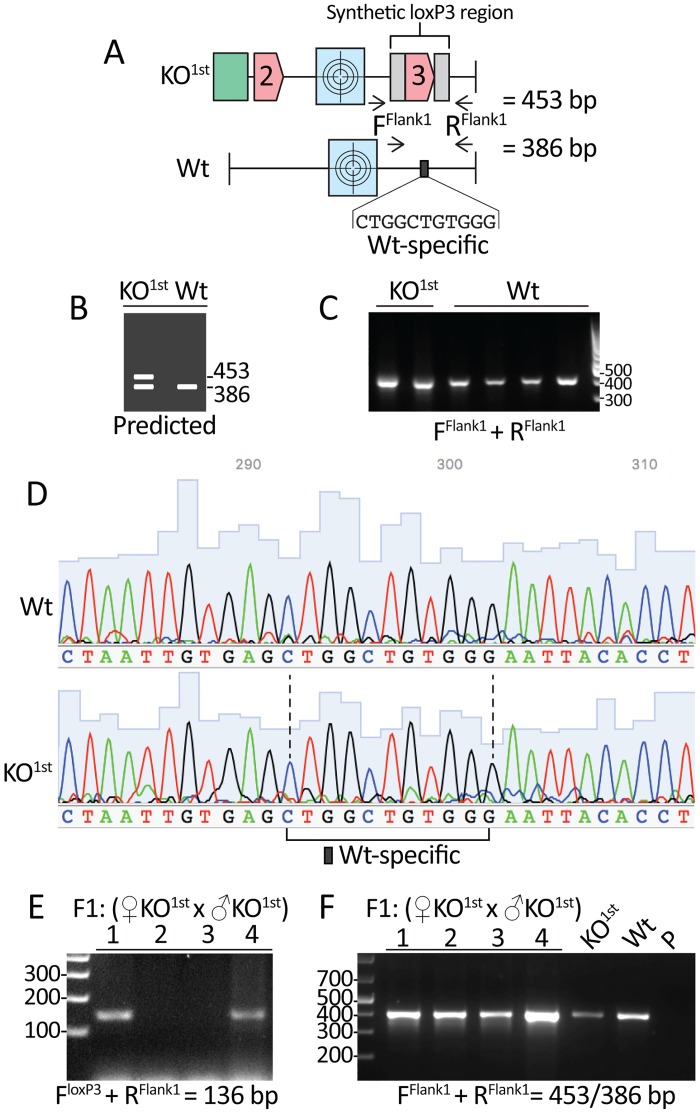
Failure to amplify templates containing the synthetic loxP3 region using flanking primers. **A.** Primers targeting Wt sequences upstream (F^Flank1^) and downstream (R^Flank1^) of the synthetic loxP3 region were predicted to amplify a PCR product of 453 bp from the KO^1st^ construct and 386 bp from Wt sequences. The 80 bp synthetic loxP3 region replaces eleven Wt-specific bps; therefore, this represents a unique identifier for amplicons derived from Wt alleles. **B.** PCR with these primers is predicted to produce 453 bp and 386 bp products from KO^1st^ chimeras and a single 386 bp product from Wt samples. **C.** A single product is amplified from KO^1st^ chimeras and Wt mice samples using the F^Flank1^ with R^Flank1^ primers. PCR was performed with an annealing temperature of 56°C and products were separated on a 1% agarose gel containing GelRed stain. **D.** Sanger sequencing of the PCR products from C confirms that only amplicons containing the Wt-specific identifier were produced. **E.** PCR with F^loxP3^ and R^Flank1^ primers using DNA isolated from four F1 progeny (derived from crossing male and female KO^1st^ chimeras) identified two individuals as positive for the KO^1st^ template. **F**. PCR with F^Flank1^ with R^Flank1^ primers failed to distinguish the KO^1st^ positive F1 progeny from Wt samples. PCR was performed with an annealing temperature of 60°C and products were separated on a 2.5% agarose gel containing GelRed stain. The two KO^1st^ lanes in C represent PCR products derived from the same two distinct KO^1st^ chimeric mice presented in Fig 1. The Wt and KO^1st^ chimera DNA samples in F are isolated from brain tissue; these samples are also used in Fig 3. *Abbreviations*: KO^1st^: Knockout first chimera, F1: Filial hybrid 1, P: Primers only.

To permit the distinction of Wt, chimeric/heterozygote and ultimately homozygote genotypes with a single PCR reaction we designed primers that flanked the synthetic loxP3 region of the *Exoc3l2*^*tm1a(KOMP)Wtsi*^ KO^1st^ construct (Fig 2A). From a practical point-of-view, the synthetic loxP3 region is the only construct-specific element that is flanked by Wt specific sequences that are sufficiently long to be targeted with primers for short range PCR. PCR with these primers was predicted to amplify a 386 bp product from Wt alleles and a 453 bp product from KO^1st^ alleles (Fig 2B). PCR of KO^1st^ and Wt biopsies using these F^Flank1^ and R^Flank1^ primers produced a single product that migrated to a point near the 400 bp marker (Fig 2C). No additional band was detected in the KO^1st^ lanes (Fig 2C). Sanger sequencing confirmed that the amplified products in KO^1st^ and Wt samples were derived from Wt templates (Fig 2D). This was evidenced by the presence of eleven Wt-specific bases that are replaced in KO^1st^ templates by the synthetic loxP3 region (Fig 2A). Therefore, while genotyping with primers that targeted elements of the KO^1st^ construct confirmed the identity of the chimeras (Fig 1), genotyping with primers flanking the synthetic loxP3 region incorrectly indicated that all samples were derived from Wt mice (Fig 2). Given the mosaic distribution of genomic material positive for KO^1st^ and Wt DNA in biopsies obtained from chimeric mice, we next addressed the possibility that the false negative results obtained using the primers flanking the synthetic loxP3 region were potentially due to low prevalence of KO^1st^ templates in the DNA samples. DNA was isolated from four female F1 progeny derived from a cross between two KO^1st^ chimeras. PCR using the F^loxP3^ and R^Flank1^ primers identified two of the four as positive for the KO^1st^ template (Fig 2E); however, once again the F^Flank1^ and R^Flank1^ primer combinations incorrectly identified all four offspring as Wt (Fig 2F). As these two F1 individuals are KO^1st^ heterozygotes the ratio of the synthetic loxP3 sequence in the DNA samples is equal to that of the equivalent Wt sequence. This suggests that the inability to amplify PCR products using primers that flank the synthetic loxP3 site does not relate to its prevalence relative to the Wt sequence.

**Fig 3:**
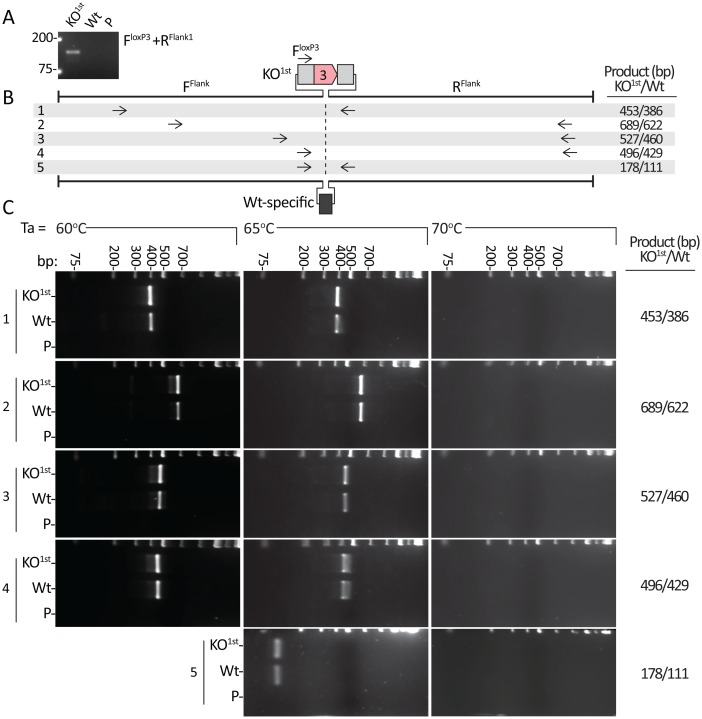
Targeting alternative flank positions or altering the PCR annealing temperature does not render templates containing the synthetic loxP3 region susceptible to amplification. **A.** PCR using the F^loxP3^ and R^Flank1^ primers correctly distinguished the genotypes of a KO^1st^ chimera (band at 136 bp) from a Wt mouse, using DNA isolated from brain tissue. **B.** Binding sites and predicted PCR product sizes for five primer pairs that target Wt sequences flanking the synthetic loxP3 region in KO^1st^ templates and an eleven bp Wt-specific sequence in Wt templates. **C.** PCR was performed with the samples from A. and the primer pairs indicated in B. with the annealing temperature (Ta) set to 60°C, 65°C or 70°C. Products were separated on 2.5% agarose gels containing GelRed stain. DNA samples from the same KO^1st^ chimeric mouse and Wt mouse were used throughout this figure. Abbreviations: P = primers only.

It is beyond the scope of this short report to elucidate the specific mechanism(s) underlying how the synthetic loxP3 region eluded amplification by PCR. However, while DMSO and betaine were included in all PCR reactions to reduce the potential formation of secondary structures, the palindromic nature of the loxP sequence encodes an inherent propensity to form secondary structures. Therefore, we considered that the failure to genotype KO^1st^ chimeras might be due to the fact that PCR with primers flanking the synthetic loxP3 region was impeded by the presence of secondary structures in the template. This would imply that the annealing temperature (Ta) of the PCR with the F^Flank1^ and R^Flank1^ primers was not high enough to melt secondary structures in the KO^1st^ template that impaired their amplification by PCR. A general recommendation for determining the Ta of a given PCR is to select a temperature approximately 5°C lower than the melting temperature of the primers. The average Tm of the F^Flank1^ and R^Flank1^ primers was 60.5°C, and the PCR was performed with a Ta of 56°C. However, Sanger sequencing detected only Wt sequences in the amplicons produced from DNA derived from KO^1st^ chimeras (Fig 2D). We next attempted to use additional flanking primers to amplify templates containing the synthetic loxP3 region by gradually increasing the Ta of the PCR. We tested five pairs of flanking primers. Their binding position relative to the synthetic loxP3 region or the Wt-specific sequence and predicted product sizes are illustrated in Fig 3B. DNA was isolated from the brains of a KO^1st^ chimera and a Wt mouse and their genotypes were confirmed using the F^loxP3^ and R^Flank1^ primers (Fig 3A). However, at a Ta of 60°C or 65°C all flanking primer pairs produced products of equal size in the KO^1st^ chimera and Wt sample, with no additional larger products observed for the KO^1st^ chimera under any conditions (Fig 3C). All primer pairs failed to amplify products in the KO^1st^ or Wt samples at a Ta of 70°C (Fig 3C), which was presumably too high to permit primer binding. Based on the results of Fig 3C alone this KO^1st^ chimera would be falsely genotyped as a Wt mouse. This suggests that only the Wt allele was amplified from the DNA isolated from the KO^1st^ chimera and that the allele carrying the synthetic loxP3 region had again eluded the PCR. Furthermore, if the synthetic loxP3 region is assuming a secondary structure that interferes with the PCR, then it remains stable at the annealing temperatures tested in Fig 3.

**Fig 4:**
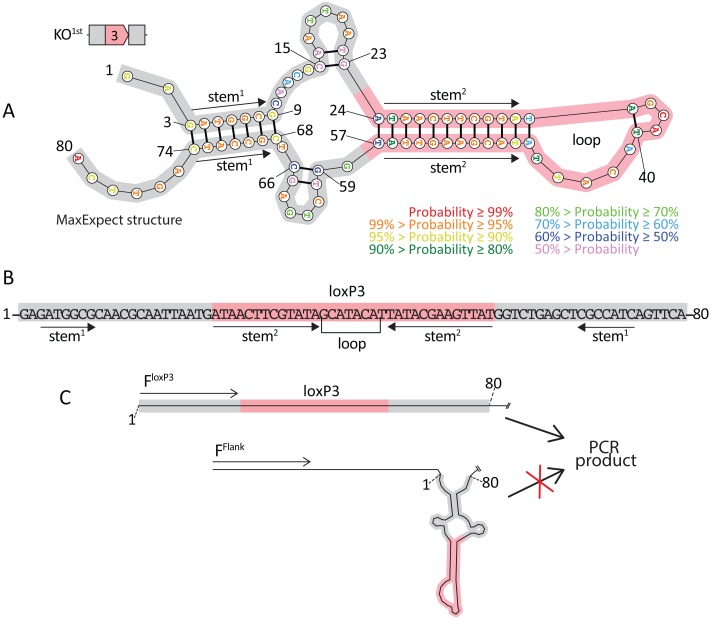
Predicted secondary structure of the synthetic loxP3 region. **A.** The 80 base synthetic loxP3 region was submitted to the RNAstructure Webserver; DNA was selected as the nucleic acid type and the default data settings were preserved. The RNAstructure MaxExpect result generated by the Webserver is presented; this represents a structure formed due to high probability base pairing. The centrally located 34 bases is the loxP sequence (highlighted in pink), of which the first and last 13 bases are palindromic sequences. This facilitates intrastrand base-pairing permitting the formation of a hairpin loop structure. An additional stem structure (stem^1^) is predicted due to intrastrand base-pairing between the synthetic DNA sequences that flank the loxP site (highlighted in grey). The probability of base-pairing is illustrated by colour coding. **B.** A linear view of the 80 bases of the synthetic loxP3 region is annotated with the position of the stem and loop structures. **C.** Primers that bind sequences that are capable of intrastrand base-pairing may compete with intrastrand base-pairing, stabilizing the template and permitting PCR to proceed. In contrast, primers that bind in the regions flanking the synthetic loxP3 region cannot prevent secondary structure formation and consequently PCR of these templates may be impaired.

The map of the *Exoc3l2*^*tm1a(KOMP)Wtsi*^ construct indicates that the 80 bp synthetic loxP3 region contains a centrally located loxP sequence (34 bases). As with all loxP sites, the first 13 bases and last 13 bases are comprised of palindromic sequences, such that they form perfect base pair matches with each other, permitting the formation of a hairpin loop secondary structure. As discussed this 80 bp sequence is specific to the KO^1st^ template and replaces eleven Wt-specific bases (Fig 2A). We submitted the 80 base sequence to the RNAstructure Webserver [8], selected DNA as the nucleic acid type, and preserved the default analysis settings. In Fig 4A we present the RNAstructure MaxExpect results, which predicts secondary structures in nucleic acid sequences based on highly probable base-pairing events. A similar structure was predicted with the RNAstructure Fold algorithm, which predicts the lowest free energy structure for a given sequence (structure not shown). High probability intrastrand base pairing is predicted between most of the two stem sequences of the loxP3 sequence (Fig 4A, bases 24–35 and 46–57). Additionally, seven high probability intrastrand base pairs are predicted between sequences in the synthetic regions flanking the loxP3 region (Fig 4A, bases 3–9 and 74–68). The position of these structures are illustrated on a linear version of the synthetic loxP3 sequence in Fig 4B. Given that PCR with the F^Flank^ and R^Flank^ primers effectively amplified Wt templates, but failed to detect their KO^1st^ counterpart (Figs 2 and 3), we propose that the propensity for secondary structure formation encoded in the synthetic loxP3 region may exclude it from the PCR, leading to false negative genotyping results. In contrast, the F^loxP3^ primer in combination with the R^Flank1^ primer was capable of amplifying its intended loxP-containing templates (Fig 1D). F^loxP3^ targets a sequence that is predicted to participate in intrastrand base-pairing (Fig 4, stem^1^); therefore, primer binding at this position may compete with intrastrand base-pairing events and in doing so stabilize the template such that it can be amplified by PCR (Fig 4C).

PCR protocols for genotyping ES cells are available on the KOMP Repository’s webpage (https://www.komp.org), several strategies are recommended including one similar in design to that described here using F^loxP2^ and R^Target^ primers (Fig 1B). The KOMP protocols also include a strategy for determining zygosity similar to that tested here with the F^Flank^ and R^Flank^ primers. They recommend that the bp size difference between the KO^1st^ and Wt amplicons is greater than 30 bp and that the amplicons themselves are less than 300 bp in size. All the F^Flank^ and R^Flank^ primers were predicted to produce amplicons that differed 67 bp between KO^1st^ and Wt alleles (Fig 3B), which are easily distinguished on a 2.5% agarose gel. It should be noted that others have successfully determined the zygosity of conditional models using similar loxP-flanking strategies (for examples see [10–13]), which amplify templates containing a loxP sequence(s). Therefore, it may be that the additional intrastrand base-pairing in the synthetic loxP3 region (Fig 4A, sequences highlighted in grey) provides greater secondary structure stability than that conferred by the loxP site (Fig 4A, highlighted in pink) alone.

## Conclusion

This report aims to highlight a source of potential false negative genotyping connected to the presence of a synthetic loxP region. BLAST alignment analysis of this 80 base pair sequence reveals that it is a common element in over 10,000 of the conditional gene-targeting mouse models available through the KOMP Repository and the European Conditional Mouse Mutagenesis Program. We hope our experience will help others in avoiding this potential source of error when devising their genotyping strategies.

## Supporting Information

S1 File Nucleotide BLAST analysis of the synthetic loxP region. Sequence alignment of the synthetic loxP region was performed using the Nucleotide Basic Local Alignment Search Tool (BLAST) hosted on the National Center for Biotechnology Information (NCBI) webserver. A summary of the results with accession identifiers for each of the transgenic mouse models that align is presented. The specific settings used for the alignment are detailed in Materials and Methods.
(XLSX)
